# Acuity and colour vision changes post intravitreal dexamethasone implant injection in patients with diabetic macular oedema

**DOI:** 10.1371/journal.pone.0199693

**Published:** 2018-06-26

**Authors:** Ahmed Abdel-Hay, Sobha Sivaprasad, Ahalya Subramanian, John L. Barbur

**Affiliations:** 1 Applied Vision Research Centre, City, University of London, London, United Kingdom; 2 Musgrove Park Hospital, Taunton, United Kingdom; 3 Moorfields Eye Hospital, London, United Kingdom; Universita degli Studi di Firenze, ITALY

## Abstract

**Purpose:**

To evaluate changes in colour vision following intravitreal injection of Dexamethasone implant (Ozurdex) in patients with diabetic macular oedema (DMO). Both red-green (RG) and yellow-blue (YB) chromatic sensitivity were assessed using the Colour Assessment & Diagnosis (CAD) test which isolates the use of colour signals and provides age-corrected, statistical limits for normal trichromats. To determine whether colour changes and visual acuity (VA) post-treatment relate to central sub-field retinal thickness (CST).

**Methods:**

Fourteen patients with DMO who were undergoing treatment with Ozurdex were recruited for this study. RG and YB colour thresholds were measured using the CAD test, best corrected visual acuity was assessed using the ETDRS chart and CST was measured using spectral domain optical coherence tomography (SD-OCT). All tests were performed monocularly at baseline and 24 weeks post injection.

**Results:**

All patients (n = 14 eyes), had significant loss of RG and YB chromatic sensitivity at baseline (p<0.05). The mean age was 56 ± 9.5 years. The age specific, monocular, upper normal limits for a 56 year old subject are 2.66 for RG and 2.85 for YB. In this study, the measured, pre injection thresholds (mean±SD) were 22.6 ± 11.3 for RG and 16.2 ± 3.76 for YB. There was significant improvement in RG threshold post injection (i.e., 19.2 ± 10.8 (p<0.05)). No significant changes were found in the YB thresholds with corresponding mean and range values of: 15.8 ± 4.6 (p = 0.23). CST pre-treatment was 542 ±135 μm. After treatment and by week 24 the CST values decreased to 435 ±127 μm.

**Conclusions:**

RG colour thresholds provide a sensitive measure of functional change in diabetic subjects with macular oedema. The YB system is damaged severely in the DMO patients studied and shows little or no recovery post treatment. The improvement in VA and particularly in RG colour vision correlate well with the measured decrease in CST. The results suggest that the improvement in the RG chromatic sensitivity can provide a useful biomarker for monitoring the efficacy of treatment in DMO.

## Introduction

Diabetic macular oedema (DMO) affects 20% of patients with DR [1] and can cause vision loss independent of the grade of retinopathy.

Inflammation plays an important role in the pathophysiology of DMO and is mediated through the expression of prostaglandins, leukotrienes and VEGF [2]. As a result, steroids can play a vital role in decreasing intracellular and extracellular oedema through inhibition of these factors by suppression of macrophage activity, vasoconstrictive effect, and reduction of lymphokine production [3].

The standard care for DMO has evolved over several years and relies mostly on the control of the systemic condition: diabetes, blood pressure and lipid management. Macular laser photocoagulation was the mainstay of treatment for over 30 years [4]. More recently anti-VEGF has been introduced for use in the treatment of DMO. Steroids can also be useful in the treatment of DMO by blocking the production of VEGF and other inflammatory mediators [5–8]. The use of steroids is, however, associated with raised intraocular pressure (IOP) in up to 50% of patients and cataract formation in 40% of the injected eyes [9]. Similar to anti- VEGF drugs such as ranibizumab, the effect is short-lived and patients require frequent injections with higher cumulative risks of side effects.

Sustained release corticosteroids have been developed with the aim of offering longer lasting effects that reduce the need for frequent intravitreal injections. The dexamethasone intravitreal implant—Ozurdex (Allergan Inc., Irvine, CA, USA) is a sustained-release biodegradable implant made of polyacticglycolic acid matrix, containing dexamethasone that is injected into the vitreous through the pars plana using a customized applicator. It releases 700mg dexamethasone slowly into the posterior segment. This treatment has been shown to be effective in chronic DMO and DMO which is resistant to anti-VEGF treatment [10, 11].).

The OCTOME study study [12] was designed to evaluated the morphological and functional changes following treatment with Ozurdex in patients with MO secondary to DR and vein occlusion. The The OCTOME report 1 confirmed that the therapeutic effect of Ozurdex lasts up to 36 weeks in terms of improvement of visual function and macular thickness in patients with MO. It also concluded that if re-treatment is needed, re-injection of Ozurdex at 20 weeks is appropriate and ensures effectiveness with least side-effects [12].

Although measurement of retinal thickness using OCT scans is a useful tool to monitor the response to treatment, it cannot substitute the measurement of VA and both OCT and VA have a place in the monitoring of diabetic changes. It is, however, well established that VA does not always correlate well with clinical severity of MO [13]. Other visual functions such as functional contrast sensitivity, colour vision (CV) and rapid flicker sensitivity may provide a better understanding of the effect of Ozurdex on MO. In this study we measured and compared changes in RG and YB colour thresholds with the corresponding changes in VA and CST to quantify the effects of Ozurdex treatment on other aspects of vision. The patients investigated in this exploratory study represent only a small subgroup recruited for the larger OCTOME study [12].

**Fig 1:**
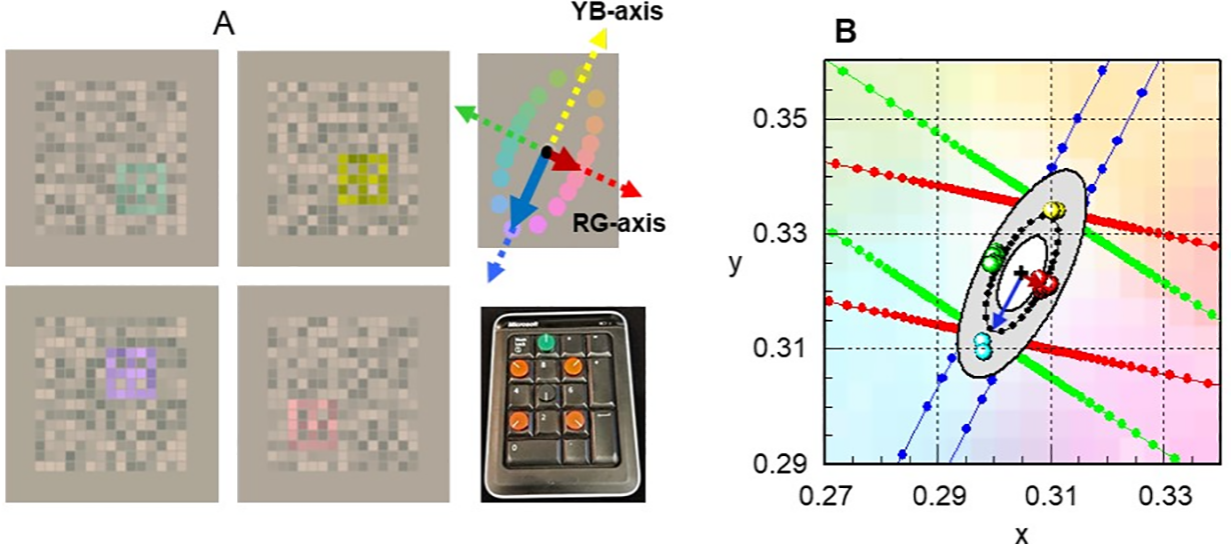
Screen dumps of CAD test stimuli and template plot. Section A shows stimulus colours that isolate the RG and YB axes. The mean threshold ellipse measured with respect to a daylight backgroud (CIE- (x, y): 0.305, 0.323) is also shown together with the RG and YB axes and the bespoke keypad employed to record the subject’s responses. Section B shows the standard CAD units for RG and YB colour vision (i.e., the ‘red’ and ‘blue’ arrows) which equal the minor and major axis of the mean threshold ellipse measured for 330 young, healthy normal trichromats [14]. The coloured sysmbols show typical thresholds measured in a subject with normal trichromatic colour vision. Section B, the grey shaded area shows the range of variation expected for young normal trichromats, the innermost and outermost ellipses corresponding to 2.5% and 97.5% confidence limits for normal population, respectively [18]. The dotted black ellipse represents the mean threshold values which define the young standard normal CAD observer. The corresponding mean thresholds are normalised to unity and all other CAD thresholds are expressed in these standard normal units (SNU). Upper normal CAD threshold limits have also been measured as a function of age in a separate study [20]. The smallest colour thresholds correspond to ~ 20 years of age and are followed by a gradual, linear increase of ~ 1% per year for RG and ~ 1.6% for YB, over the remaining life span. Each patient’s thresholds measured in this study were compared against the upper normal threshold limits for the corresponding age [20].

Our specific aim was to measure and compare changes in RG and YB thresholds using the Colour Assessment and Diagnosis (CAD) test [14] “Fig 1” in patients with DMO pre- and post-Ozurdex (Dexamethasone implant) intravitreal injection. The study assessed the severity of RG and YB colour loss before treatment and established whether treatment with Ozurdex led to any significant changes in colour vision. Of particular interest is to establish whether selective changes in RG and / or YB colour vision correlate with reduction in CST and whether such changes can be used to evaluate the efficacy of Ozurdex treatment in DMO.

## Methods

Subjects were recruited from the Ophthalmology outpatient department of King’s College Hospital NHS Foundation Trust, London. The study was approved by the Research and Ethics Committees of King’s College Hospital and City, University of London. All participants gave written informed consent and the study adhered to the principles of the Declaration of Helsinki.

### Subjects

This study examined 14 diabetic patients with DMO. The participants represent a subset recruited from the 24 diabetic patients examined in the OCTOME study which was an interventional, prospective and exploratory study [12]. The remaining 10 diabetic patients, not included in this study, had either age-related decline in cognitive ability or very poor visual acuity. As a result they were unable to complete the one minute learning stage with 100% accuracy, a task which does not rely on the use of CV. During the learning task, the CAD stimulus “Fig 1” is defined by both colour and luminance contrast and is always seen by both normal trichromats and dichromats. 100% performance in the learning task is recommended to ensure that the patients understand the requirements of the visual task and can use the keypad response buttons “Fig 1” without error.

All participants had to meet the criteria listed below:

- The best corrected visual acuity (BCVA) in the study eye had to be in the range 37 and 68 ETDRS Letters, this level of performance is equal to 1.26 and 0.64 LogMAR acuity, respectively.
- Patients were excluded if they had any other eye disease which could mask or contribute to MO, or any ocular condition in the study eye that would prevent a 15-letter improvement in visual acuity (e.g., severe macular ischemia, extensive macular laser scarring or atrophy).
- Patients were also excluded if they had advanced glaucoma that was not controlled adequately by drugs alone, or a history of IOP elevation in response to steroid treatment in either eye that resulted in ≥10mmHg increase in IOP from baseline with an absolute IOP ≥25mmHg, or required therapy with three or more anti-glaucoma medications.
- Patients were excluded if they had any systemic conditions that precluded trial entry such as known uncontrolled systemic disease or current immunosuppressive disease, initiation of medical therapy for diabetes, or a change from oral hypoglycaemic agents to insulin therapy within 4 months before the screening visit and renal failure requiring haemodialysis or peritoneal dialysis within 6 months before screening visit.

### Ophthalmic assessments

All patients were examined at baseline (week 1) and then subsequently at 24 weeks for this arm of the OCTOME study. Monocular BCVA was measured using the standard ETDRS protocol and scored as the total number of ETDRS letters read correctly. CST was measured using the Spectralis SD-OCT (Heidelberg engineering GmbH, Heidelberg, Germany). CST measurements are generated automatically and follow the 9^th^ Early Treatment Diabetic Retinopathy Study (ETDRS) subfields which are arranged in inner, intermediate and outer rings with radii of 1mm, 2.22mm and 3.45 respectively. The average of all points within the inner circle is defined as central sub-field thickness. Monocular RG and YB chromatic sensitivity was measured with the CAD test [14].

The CAD test (City Occupational Ltd., London, UK) measures colour thresholds along 16 directions, away from a ‘white’ background equivalent to daylight (D_65_). The measured thresholds provide an accurate estimate of the severity of RG and YB colour vision loss. The CAD results also classify the participant’s class of colour vision (i.e., normal trichromatic colour vision, deutan-, protan-, tritan-like congenital deficiency or acquired deficiency).

The stimulus is generated with 30 bit resolution in the centre of a large uniform background field on a visual display (NEC-spectraview PA241-W). The coloured stimulus is buried in dynamic luminance contrast noise and subtends ~ 30 x 30 min arc at the eye “Fig 1” from a recommended viewing distance of 1.4m. The test is based on background perturbation techniques [15–18] developed to mask detection of luminance contrast and to isolate the use of colour signals [14, 19].

Patients with refractive errors wore their appropriate refractive correction for the corresponding distance of 1.4 m. All patients viewed the visual display with the head / eye position in line with the centre of the monitor. The test was carried out monocularly in the eye with the best BCVA. The full CAD test takes about 3 minutes for YB and ~ 10 minutes for RG to complete.

The initial validation of the CAD test and the statistical limits needed to describe RG and YB colour sensitivity in young subjects with normal trichromatic colour vision are based on RG and YB thresholds measured in 330 normal trichromats (Civil Aviation Authority (UK), 2009).

### Optical coherence tomography

OCT scans were carried out using the standard protocol on the Heidelberg Spectralis SD-OCT (Heidelberg Engineering, Heidelberg, Germany) [21]. All scans were performed by two experienced medical photographers.

All patients had the Ozurdex (Dexamethasone implant) intravitreal injection on week 1 (baseline). According to the protocol from OCTOME study some of the patients were eligible for a second injection at the re-treatment window between week 16 and week 24.

Changes in the patient’s VA, RG and YB thresholds were compared against the corresponding changes in CST.

## Statistical analysis

The data were analysed using MS Excel (V.15.0) and SPSS programs (SPSS V.22.0, SPSS, Chicago, IL, USA). Analysis was performed using t-tests to calculate p values and categorical variables were examined using Pearson’s r^2^ correlation. Differences in results were assumed significant for p-values <0.05.

## Results

### Descriptive data

The study included 14 patients. Demographic and baseline characteristics are shown in Table 1.

**Table 1:** Demographic and baseline characteristics of the patients examined in this study.

Mean age (SD), years			56±9.23
Range, years			40–68
*Gender*, *n (%)*			
	Male, *n* (%)		12 (85.7)
	Female, *n* (%)		2 (14.2)
*Race*, *n (%)*			
	Afrocaribbean		4 (28.6)
	Asian		2 (14.3)
	White		8 (57.1)
*Grade of diabetic retinopathy*, *n (%)*			
	Mild and moderate NPDR		6 (42.8)
	Severe NPDR		3 (21.4)
	Treated PDR		5 (35.7)
Smokers, *n* (%)			2 (14.3)
*Mean HbA1C*, *n (SD)*			7.8±1.39
	≤8%, *n* (%)		6 (50)
	>8%, *n* (%)		6 (50)
Hypertensives on treatment, *n* (%)			9 (64.3)
Previous glaucoma medications			0
Previous macular laser, *n* (%)			12 (85.7)
Mean number of laser treatments, *n* (SD)			2.2±1.6
*Mean ETDRS letter score*, *n (SD)*			59.71±9.20
	<54 letters, *n* (%)		4 (28.6)
	≥54 letters, *n* (%)		10 (71.4)

Abbreviations: ETDRS (early treatment diabetic retinopathy study); HbA1C (glycosylated haemoglobin); NPDR (non-proliferative diabetic retinopathy); PDR (proliferative diabetic retinopathy)

### Lens opacity

Lens yellowing and senile pupil miosis have been linked to age-related deterioration of CV caused largely by significant reduction in retinal illuminance [22, 23]. The density of cataract in the current study was graded using Lens Opacities Classification System III (LOCS III) [24] “Table 1”. 12 patients had LOCS III grading ≤ N1C1 and two patients were pseudophakic.

### Change in RG and YB thresholds

**Fig 2:**
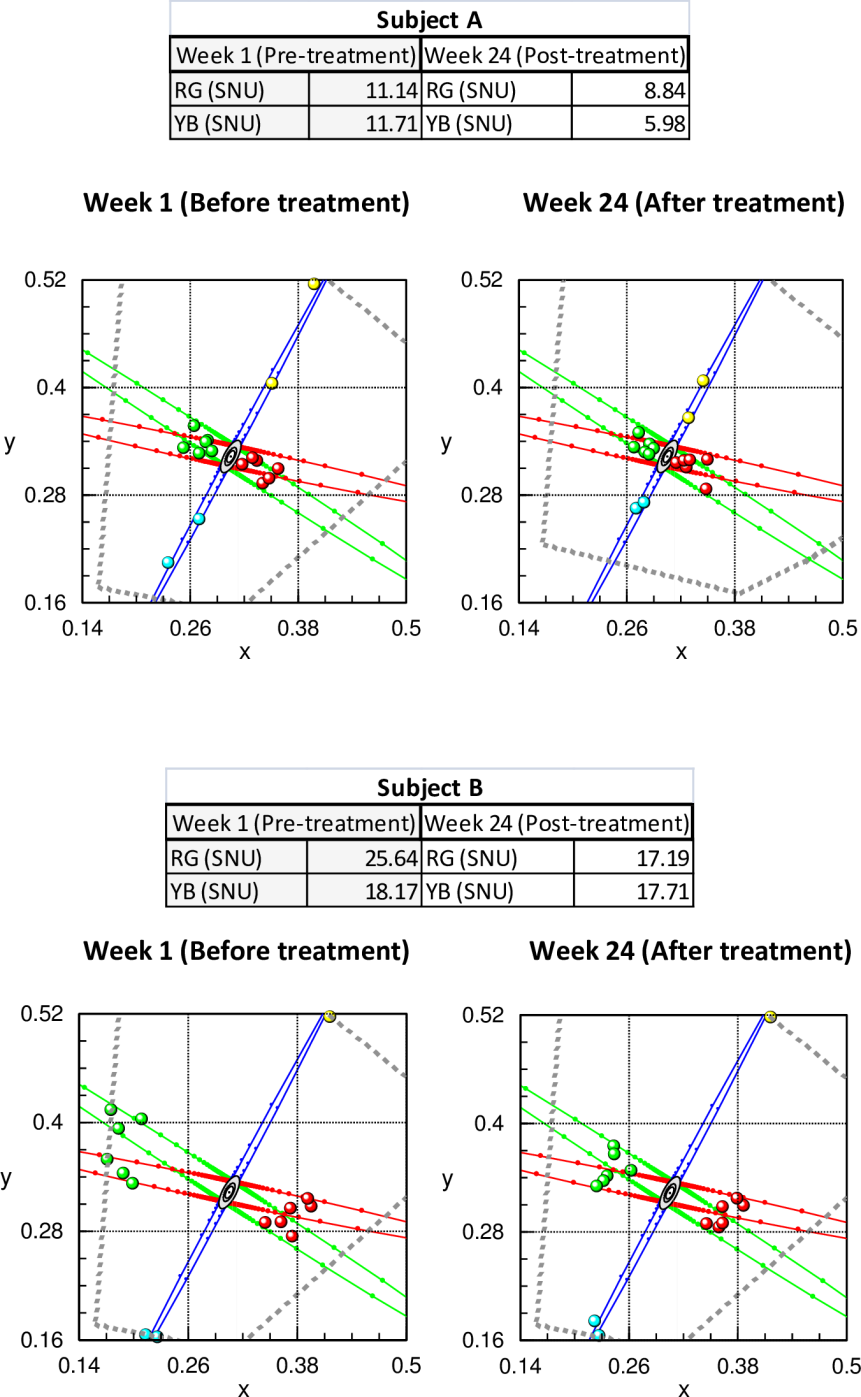
**CAD results before and after Ozurdex treatment in two subjects with diabetes (A- subject 11; B- subject 4).** Both subjects show significant improvement in chromatic sensitivity (i.e., smaller thresholds) post treatment. The grey, dotted lines show the colour limits imposed by the phosphors of the display. Subject B was unable to detect YB colour changes, even for the largest chromatic signals that are limited only by the phosphors of the display with no improvement post treatment. The RG thresholds, on the other hand, show significant improvement post-treatment.

The mean age of the 14 patients was 56 years (range, 40 to 68) and the age specific, monocular, upper normal CAD limits for a 56 years old subject are 2.66 for RG and 2.85 for YB. In this study, all the patients were outside the normal, age-matched, upper threshold limits [20]. The RG pre-injection threshold was 22.57 ±11.27 (range, 2.95 to 35.87) whilst the YB mean threshold was 16.21 ± 3.76 (range, 15.18 to 18.19) “Fig 2”. It is important to note that the upper limit of 18.19 CAD units was the highest possible chromatic displacement along the tritanopic colour confusion line.

**Fig 3:**
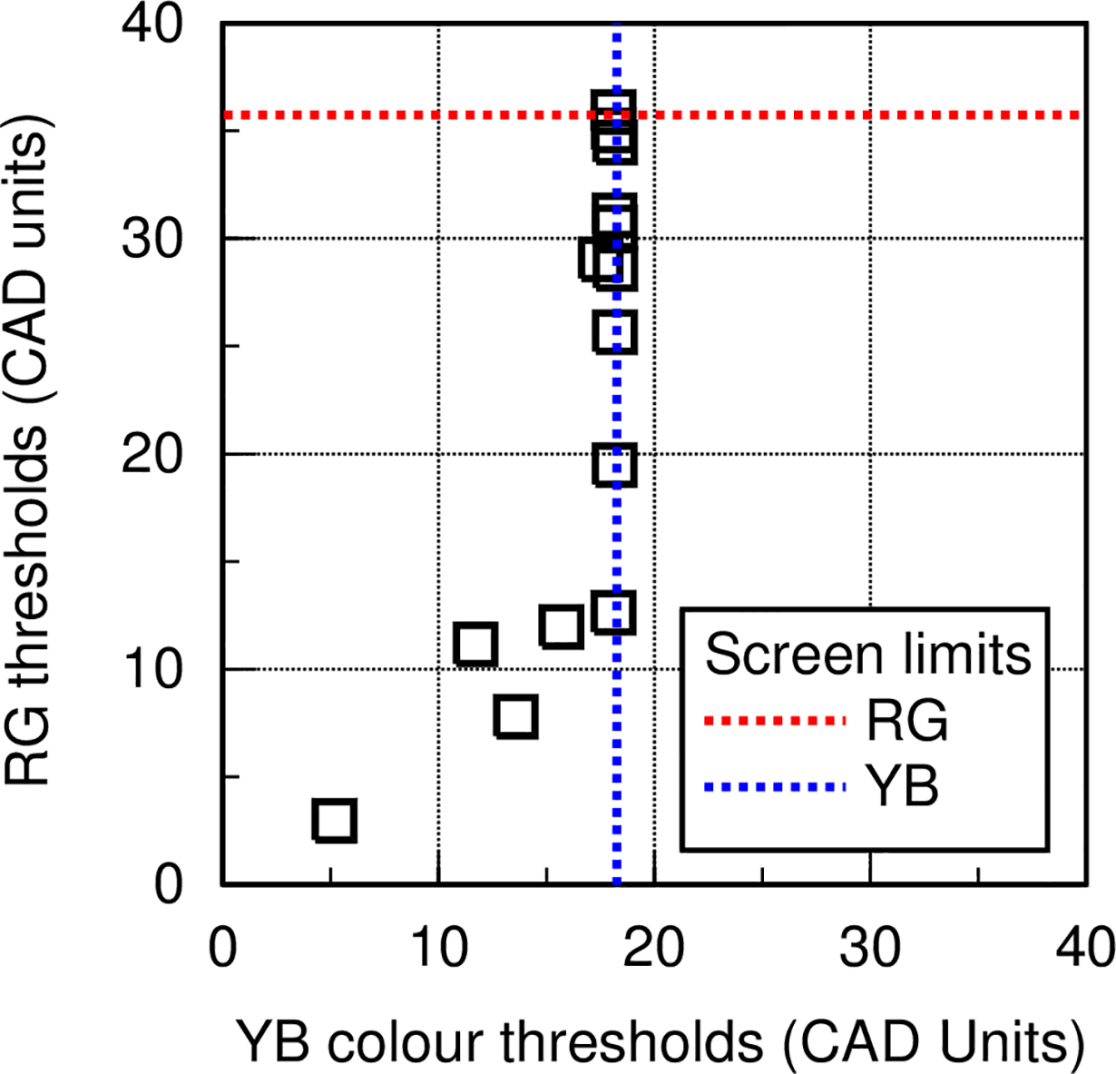
RG and YB thresholds. Shows thresholds measured before treatment in the 14 DMO patients, together with the maximum chromatic saturations that can be produced on the visual display (i.e., the limits of colour saturation in CAD units that can be generated on the visual display).

Significant improvements in RG thresholds post injection 19.18 ±10.84 (t (13) = 1.965, p<0.05) were observed. Post injection the YB thresholds were only fractionally smaller with reductions that failed to reach statistical significance (15.84 ± 4.60 (t (13) = 0.747, p = 0.23). Only three patients showed significant improvement in their YB threshold “Table 2 and Fig 3”. The results shown in “Fig 3” are of interest since 11 out of the 14 patients have severe loss of YB colour vision with thresholds that are limited only by the visual display. The damage to the YB system is therefore extensive and these subjects show little or no recovery of YB sensitivity post treatment. In contrast, 11 out the 14 patients studied have RG thresholds that are well below the display limits and show significant recovery post treatment.

**Table 2:** Change in RG and YB thresholds, visual acuity and CST.

Subject	SNO R-G			SNO Y-B			BCVA ETDRS Letters (LogMAR)			CST		
	Before	After	*Change*	Before	After	*Change*	Before	After	*Change*	Before	After	*Change*
**1**	31.07	30.30	-0.77	18.15	18.10	-0.05	61 (0.48)	59 (0.52)	-2	530	411	-119
**2**	19.46	22.99	3.53	18.16	18.08	-0.08	67 (0.36)	85 (0.00)	18	580	411	-169
**3**	12.61	15.93	3.32	18.10	18.07	-0.03	68 (0.34)	59 (0.52)	-9	491	533	42
**4**	25.64	17.20	-8.44	18.17	17.71	-0.46	67 (0.36)	76 (0.18)	9	501	378	-123
**5**	34.45	34.58	0.13	18.18	18.18	0.00	40 (0.90)	39 (0.92)	-1	422	518	96
**6**	7.78	9.63	1.85	13.58	16.95	3.37	58 (0.54)	64 (0.42)	6	572	603	31
**7**	28.58	22.54	-6.04	18.19	18.13	-0.06	52 (0.66)	71 (0.28)	19	685	692	7
**8**	11.96	8.95	-3.01	15.70	14.28	-1.42	67 (0.36)	73 (0.24)	6	585	385	-200
**9**	35.87	36.18	0.31	18.10	18.16	0.06	65 (0.40)	64 (0.42)	-1	578	507	-71
**10**	30.51	17.72	-12.79	18.18	18.15	-0.03	49 (0.72)	69 (0.32)	20	573	299	-274
**11**	11.14	8.84	-2.30	11.72	5.99	-5.73	58 (0.54)	70 (0.30)	12	390	448	58
**12**	35.02	31.82	-3.20	18.12	18.05	-0.07	68 (0.34)	77 (0.16)	9	315	235	-80
**13**	29.03	9.57	-19.46	17.52	17.43	-0.09	48 (0.74)	73 (0.24)	25	881	268	-613
**14**	2.95	2.35	-0.60	5.18	4.57	-0.61	68 (0.34)	75 (0.20)	7	485	414	-71

### Individual changes in VA, CST and colour thresholds

**Fig 4:**
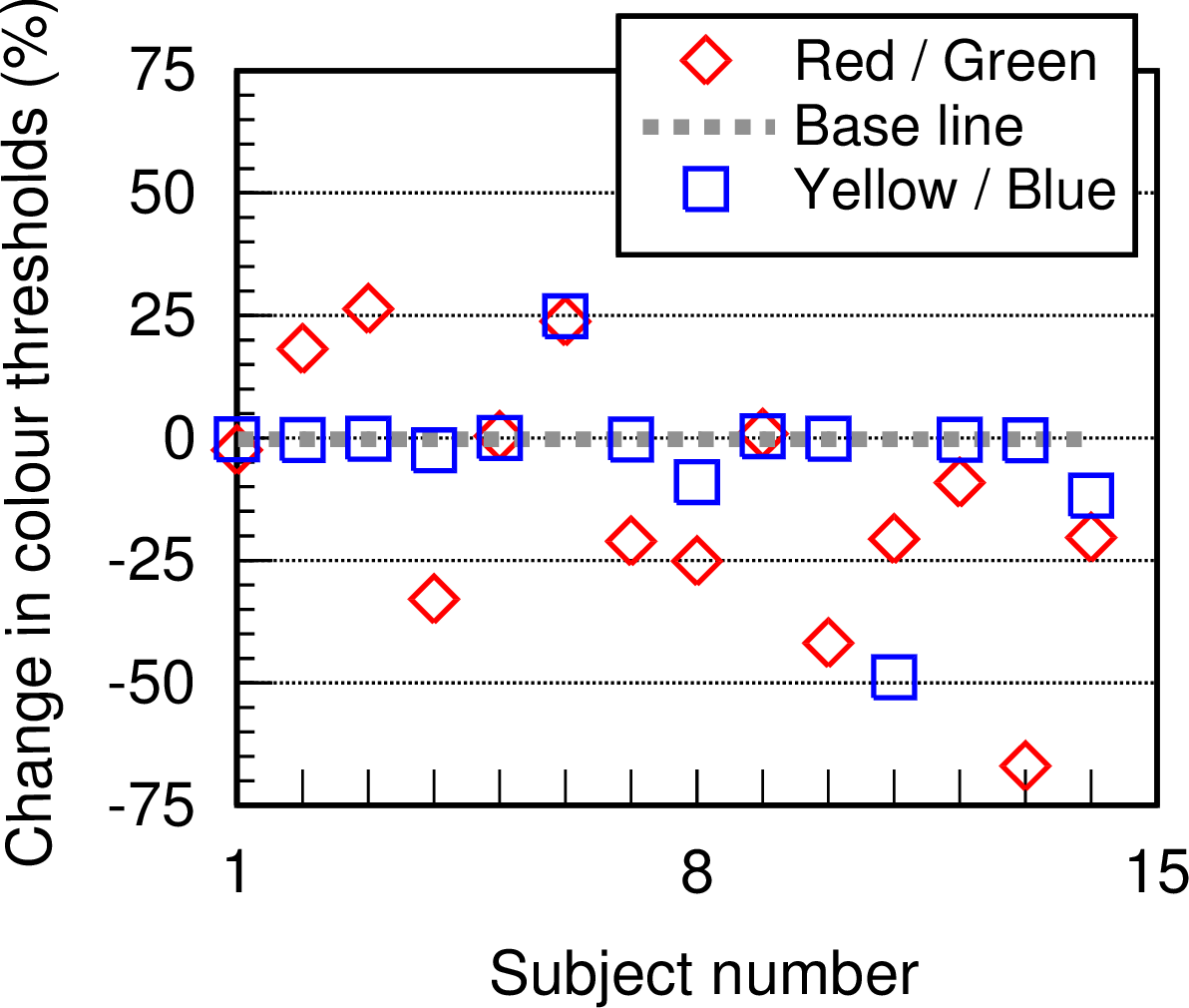
Changes in RG and YB colour thresholds following treatment with Ozurdex, plotted for each of the patients examined. A ‘negative’ percentage change indicates an improvement. It shows little or no improvement in YB colour vision, but a significant improvement in RG colour vision in the majority of patients.

The benefits of Ozurdex treatment is calculated by measuring the patient-specific changes in RG and YB colour thresholds, VA and CST at week 24, post treatment. The data for RG and YB colour vision are shown in “Fig 4”. A negative change represents improvement. There was little or no improvement in YB colour vision in all but one patient post treatment. The YB colour system is, however, heavily damaged in this patient group with thresholds limited only by the visual display “Fig 3”. This is not the case for RG thresholds, VA and CST, all of which show significant improvements in the majority of patients post treatment.

### CST changes and corresponding correlations with colour vision and VA

**Fig 5:**
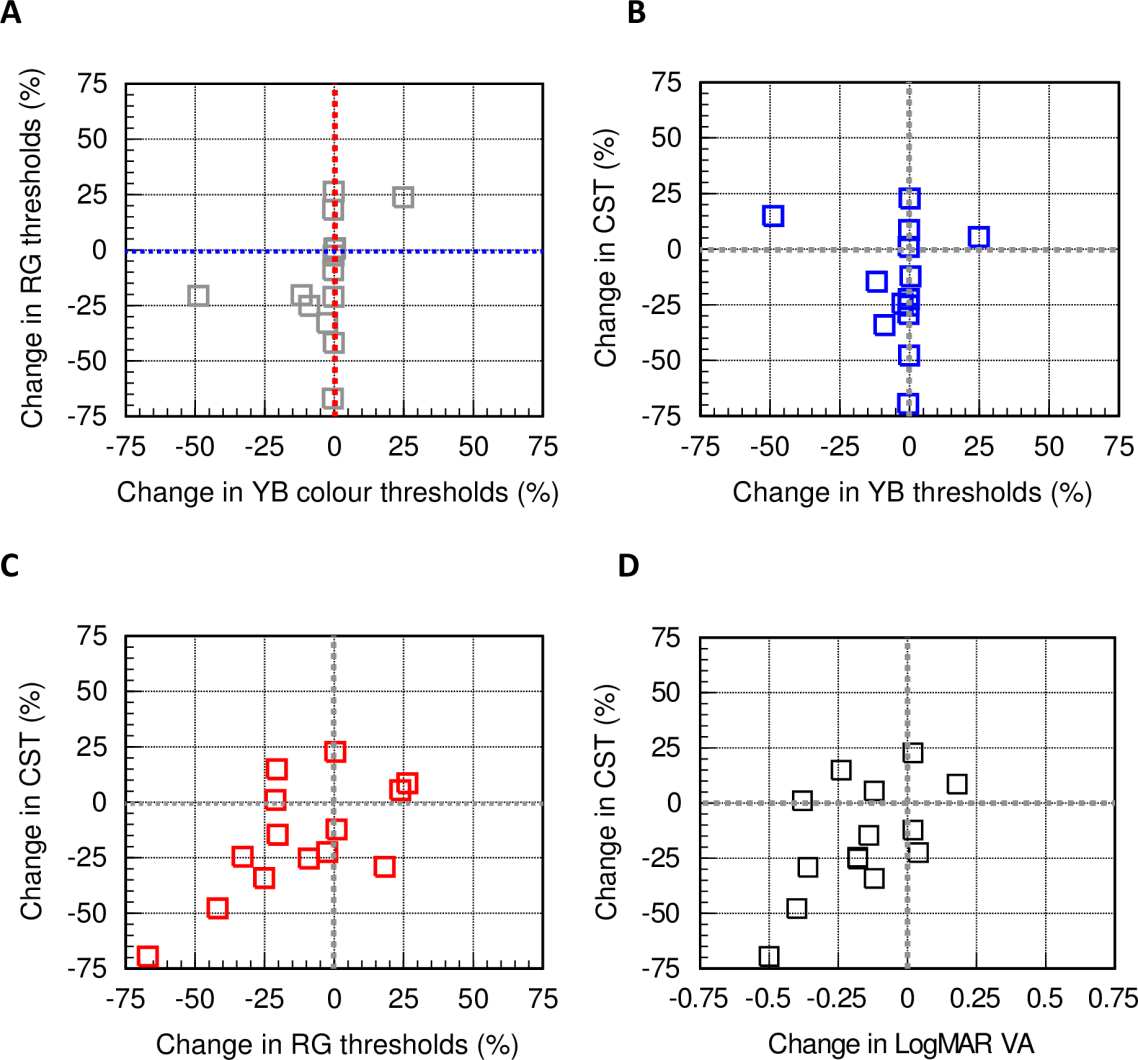
CST changes and corresponding correlations with colour vision and VA. Section A illustrates more clearly the absence of significant changes in YB thresholds when compared to the improvements in RG colour vision. Sections B and C show how the percentage decrease in CST correlates with the corresponding changes in YB (B) and RG (C) thresholds. Section D shows how changes in CST correlate with the changes in LogMAR VA post treatment. A ‘negative’ change indicates an improvement in VA. The results suggest that decreased CST values correlate well with improvement in RG colour vision (r^2^ = 0.43) and VA (r^2^ = 0.34). The YB system, on the other hand, appears to be damaged more severely and shows negligible correlation with the corresponding CST changes (r^2^ = 0.05).

The changes in RG colour vision are also compared for each subject against the corresponding YB changes “Fig 5(A)”. The data show clearly that whilst the majority of patients show significant improvement in RG thresholds, with one exception, the YB thresholds remain largely unchanged post treatment. The results also demonstrate the importance of CST measurements in patients with DMO. With the exception of the YB system which may be permanently damaged in many of the patients examined in this study, the extent of recovery in both VA and RG colour vision correlates well with differences in CST measurements “Fig 5C and 5D”. Percentage differences in RG thresholds exhibit the highest correlation with CST “Fig 5C”. Individual patient data are also shown in Table 2. The number of patients who gained more than 5 or 15 letters by week 24 were 6 (43%) and 4 (28%), respectively. Three patients (21%) lost 2 letters or less, with another patient losing 9 letters by week 24. The mean CST measured before treatment with Ozurdex was 542 ±135 μm. Following treatment and by week 24 the mean CST was found to be 435 ±127 μm, a significant reduction consistent with the percentage improvements in RG thresholds “Fig 5C”, (t (13) = 2.202, p<0.05).

## Discussion

This preliminary study examined changes in RG and YB chromatic sensitivity, VA and CST in eyes undergoing intravitreal injection of dexamethasone implant (Ozurdex) as treatment for DMO. All patients showed loss of RG and YB chromatic sensitivity at recruitment with YB colour vision being most affected “Fig 3”. Recovery of YB colour vision post treatment “Figs 4 and 5A” is almost absent and this suggests significant damage to YB chromatic mechanisms in all but one of the DMO patients. RG sensitivity, on the other hand, recovers well post treatment “Fig 5A”. The differences in RG sensitivity between post and pre-treatment tests correlate well with the corresponding individual reduction in CST. This observation also holds true for differences in VA, although higher correlation is observed with differences in RG thresholds “Fig 5”.

All patients recruited in our study had chronic DMO, therefore they would have had previous treatments such as laser—focal or grid, intravitreal triamcinolone and anti-VEGF injections. Given the advanced level of damage to the retina, it was not unexpected to find significant loss of colour vision in all patients with YB colour vision being almost absent in some patients. Most of the patients examined had a degree of cataract which is a known association with diabetes [25]. This can affect chromatic sensitivity when the ambient light level is low causing an overall reduction in retinal illuminance. Although ocular opacities may have contributed in part to the massive loss of YB chromatic sensitivity, the absence of significant recovery in YB thresholds in all but one of the patients “Fig 5A” suggests the presence of significant permanent damage to the YB system. The RG system shows greater resilience in DMO with significant post treatment recovery which correlates well with the reduction in retinal thickness “Fig 5C”.

VA also improves significantly post treatment, but the correlation with percentage differences in CST is highest for RG colour vision “Fig 5C and 5D”.

In the related study-OCTOME [12], Ozurdex produced an improvement in visual acuity with maximal gain achieved by 12 weeks. At 24 weeks post treatment, while the gain in visual acuity started to regress, RG colour vision continued to exhibit significant improvement. Interestingly, the mean decrease in CST showed a similar trend with initial significant improvement that was more evident by week 8, followed by smaller changes from week 16 onwards.

The data from this preliminary study suggest that changes in CST thickness correlate best with differences in RG colour vision followed by VA. The results also suggest that the YB system can be damaged permanently in DMO with little or no recovery post treatment.

Many previous studies have established that chromatic sensitivity is affected in diabetic retinopathy [26–29]. The mechanisms by which colour vision is affected in DMO remain poorly understood. Some explanations have been put forward to account for the observed losses, but these have been questioned. Several studies [30–33] examined the events of vascular and neuronal apoptosis that occur in DR as the prime cause of colour vision loss. These studies above focused on the various mechanisms that are thought to cause retinal cell apoptosis (vascular and neuronal retina). Exposure to oxidative stress and reduced growth factor signalling have been identified as potential contributors to loss of colour vision. Hyperglycaemia can induce oxidative stress leading to apoptotic signalling. Growth factor signalling is essential for the survival of neurones, pericytes and endothelial cells. These studies demonstrated how diabetes impairs the trophic signals pathway leading to reduction in survival signals and hence increased chances of apoptosis.

Barber at al., [34] also demonstrated that glutamate excitotoxicity can cause damage to the neuronal retina and therefore contribute to both chronic and acute neurodegeneration. Neuro-inflammation is also an important factor to be considered in the pathology of diabetic retinopathy and subsequent colour vision defects. Some studies [35–37] have identified increased levels of cytokines, especially vascular endothelial growth factor (VEGF), interleukin (IL)-1 B, IL-6, IL-8 and tumour necrosis factor (TNF)-α in the vitreous of DMO patients and patients with PDR.

The effect of circulating oxygen saturation on colour thresholds has also been investigated [38]. Dean et al., examined 37 Type I diabetics with either mild or absent DR. An improvement in colour thresholds was demonstrated in all subjects after breathing oxygen (100%). They concluded that reduced oxygen saturation can lead to impaired colour vision which is likely to be because photoreceptors are functioning at suboptimal oxygen levels. A similar study conducted by Connolly et al., [39] showed that chromatic sensitivity was impaired in aircrew when subjected to mild hypoxia as opposed to those under normoxic or hyperoxic conditions.

A majority of our patients showed significant improvement in the morphology of the macula on OCT scan at one month following their injection. This initial improvement in visual acuity and CST regressed slightly in the following weeks, but the initial improvement in chromatic sensitivity could be demonstrated during week 24.

The almost immediate fluid drying effect of the Ozurdex on the macula with restoration of anatomical structure can be seen in OCT images. This does not, however, reflect the extent of damage sustained in the neural retina and can affect some aspects of visual performance such as the more permanent loss of YB colour vision. In order to assess the full extent of damage caused by diabetes, the measurement of other visual attributes becomes of interest. Assessment of chromatic sensitivity changes as shown in this study can have an important role in assessing the progress of diabetes and the effectiveness of treatment.

DMO is the major contributing factor to loss of sight in DR. It is therefore important to detect and monitor changes in foveal vision during the course of diabetes. In addition, it is equally important to be able to detect and monitor how visual performance changes during the course of treatment so as to be able to assess objectively the effectiveness of treatment.

The findings from this study suggest that RG threshold quantified with the CAD test can be used to monitor treatment of DMO. At base line all our patients showed loss of RG and YB thresholds, but post injection with Ozurdex nine out of 14 patients showed significant improvement in RG thresholds whilst YB colour vision showed little or no improvement.

The current findings are consistent with the outcome of the OCTOME study in relation to the overall improvement in visual functions post Ozurdex injection. The findings from the OCTOME study “indicate a positive effect on the retinal neuronal function, probably due to a realignment of neuronal structures induced by the drying effect of Ozurdex” [12]. The significant improvement in RG colour vision observed in this study supports this claim and is consistent with the view that ‘A direct neurotrophic effect of Ozurdex cannot be ruled out‘, provided the treatment is administered early to avoid permanent damage, as may well be the case with the less resilient YB system.

To our knowledge this is the first study to examine the effect of Ozurdex intravitreal injection on chromatic sensitivity. It may be of great benefit in the future to conduct a similar study to investigate changes in chromatic sensitivity, contrast sensitivity and rapid flicker in naïve patients with DMO who are receiving Ozurdex as their first line of treatment. Such a study may reveal the full functional benefits of Ozurdex treatment and not just the improvement in BCVA and macular morphology. A new high–sensitivity CAD screener is currently being developed which will enable individuals to test for both acquired and congenital loss of colour vision allowing self-monitoring at home.

As per the protocol of the OCTOME study the CAD test was only done once after the injection, at week 24, but it may be useful in future studies to examine the patients again beyond week 24 to find out if the observed improvement in RG colour thresholds is sustained.

**In conclusion,** the major findings from this study reveal significant improvement in RG colour thresholds post treatment with Ozurdex, with little or no improvement in YB thresholds. Both RG chromatic sensitivity and VA improve post treatment but the percentage improvements in RG colour vison correlate best with differences in CST “S1 Table”. The findings from this study suggest that in addition to VA and CST, the recovery of RG chromatic sensitivity can be a useful biomarker in monitoring the efficacy of treatment in DMO.

## Supporting information

S1 Table The data collected for the 14 patients included in the study. It shows data pre and post injection of intravitreal Dexamethasone implant.
(XLSX)

## References

[1] Yau J.W., Rogers S.L., Kawasaki R., Lamoureux E.L., Kowalski J.W., Bek T., et al Global prevalence and major risk factors of diabetic retinopathy. Diabetes care, 2012; 35 (3) pp.556–564. doi: 10.2337/dc11-1909 22301125 10.2337/dc11-1909 PMC3322721

[2] Bhagat N., Grigorian R.A., Tutela A. and Zarbin M.A. Diabetic macular edema: pathogenesis and treatment. Survey of ophthalmology, 2009; 54 (1) pp.1–32. doi: 10.1016/j.survophthal.2008.10.001 19171208 10.1016/j.survophthal.2008.10.001

[3] Abe T., Hayasaka S., Nagaki Y., Kadoi C., Matsumoto M. and Hayasaka Y. Pseudophakic cystoid macular edema treated with high-dose intravenous methylprednisolone. Journal of cataract and refractive surgery, 1999; 25 (9) pp.1286–1288. 10476516 10.1016/s0886-3350(99)00159-5

[4] 'Photocoagulation for diabetic macular edema. Early Treatment Diabetic Retinopathy Study report number 1. Early Treatment Diabetic Retinopathy Study research group. Archives of ophthalmology, 1985; 103 (12), pp.1796–1806. 2866759

[5] Nguyen Q.D., Brown D.M., Marcus D.M., Boyer D.S., Patel S., Feiner L., et al Ranibizumab for diabetic macular edema: results from 2 phase III randomized trials: RISE and RIDE. Ophthalmology, 2012; 119 (4) pp.789–801. doi: 10.1016/j.ophtha.2011.12.039 22330964 10.1016/j.ophtha.2011.12.039

[6] Jonas J.B., Kreissig I., Sofker A. and Degenring R.F. Intravitreal injection of triamcinolone for diffuse diabetic macular edema. Archives of Ophthalmology, 2003; 121 (1) pp.57–61. 12523885

[7] Ip M.S., Bressler S.B., Antoszyk A.N., Flaxel C.J., Kim J.E., Friedman S.M., et al A randomized trial comparing intravitreal triamcinolone and focal/grid photocoagulation for diabetic macular edema: baseline features. Retina (Philadelphia, Pa.), 2008; 28 (7) pp.919–930. 10.1097/IAE.0b013e31818144a7 PMC2796075 18698292

[8] Gillies M.C., Simpson J.M., Gaston C., Hunt G., Ali H., Zhu M., et al Five-year results of a randomized trial with open-label extension of triamcinolone acetonide for refractory diabetic macular edema. Ophthalmology, 2009; 116 (11), pp.2182–2187. doi: 10.1016/j.ophtha.2009.04.049 19796823 10.1016/j.ophtha.2009.04.049

[9] Loewenstein A. and Goldstein M. Intravitreal triamcinolone acetonide for diabetic macula edema. The Israel Medical Association journal: IMAJ, 2006; 8 (6) pp.426–427. 16833175

[10] Kuppermann B.D., Blumenkranz M.S., Haller J.A., Williams G.A., Weinberg D.V., Chou C., et al Randomized controlled study of an intravitreous dexamethasone drug delivery system in patients with persistent macular edema. Archives of Ophthalmology, 2007; 125 (3) pp.309–317. doi: 10.1001/archopht.125.3.309 17353400 10.1001/archopht.125.3.309

[11] Boyer D.S., Yoon Y.H., Belfort R. Jr., Bandello F., Maturi R.K., Augustin A.J., et al Three-year, randomized, sham-controlled trial of dexamethasone intravitreal implant in patients with diabetic macular edema. Ophthalmology, 2014; 121 (10) pp.1904–1914. doi: 10.1016/j.ophtha.2014.04.024 24907062 10.1016/j.ophtha.2014.04.024

[12] Mathew R., Pearce E., Muniraju R., Abdel-Hay A. and Sivaprasad S. Monthly OCT monitoring of Ozurdex for macular oedema related to retinal vascular diseases: re-treatment strategy (OCTOME Report 1). Eye (London, England), 2014; 28 (3) pp.318–326. 10.1038/eye.2013.287 PMC3965819 24384961

[13] Diabetic Retinopathy Clinical Research Network, Browning D.J., Glassman A.R., Aiello L.P., Beck R.W., et al Relationship between optical coherence tomography-measured central retinal thickness and visual acuity in diabetic macular edema. Ophthalmology, 2007; 114 (3) pp.525–536. doi: 10.1016/j.ophtha.2006.06.052 17123615 10.1016/j.ophtha.2006.06.052 PMC2585542

[14] Barbur J. L. and Connolly D. M. Effects of hypoxia on color vision with emphasis on the mesopic range. Expert Rev. Ophthamol., 2011; 6, 409–420.

[15] Barbur J.L. and Ruddock K.H. Spatial characteristics of movement detection mechanisms in human vision. II. Chromatic stimuli. Biological cybernetics, 1980; 37 (2), pp.93–98. 7397283 10.1007/BF00364248

[16] Barbur J. L., Holliday I. E. and Ruddock K. H. The spatial and temporal organisation of motion perception units in human vision. Acta Psychol (Amst), 1981;48, 35–37. 7304238 10.1016/0001-6918(81)90046-9

[17] Barbur J. L., Harlow A. J. and pant G. T. Insights into the different exploits of colour in the visual cortex. Proc Biol Sci, 1994; 258, 327–34. doi: 10.1098/rspb.1994.0181 7886066 10.1098/rspb.1994.0181

[18] Barbur, J.L.<, Rodriguez-Carmona, M., Harlow, J.A. Establishing the statistical limits of normal chromatic sensitivity CIE Proceedings; 75 years of the Standard Colorimetric Observer, May 2006 Ottawa, Ontario.

[19] Barbur J.L. Double-blindsight' revealed through the processing of color and luminance contrast defined motion signals. Progress in brain research, 2004; 144 pp.243–259. doi: 10.1016/S0079-6123(03)14417-2 14650853 10.1016/S0079-6123(03)14417-2

[20] Barbur J. L. and Rodriguez-Carmona M. Color vision changes in normal aging. E. A. J., F. M.D. and F. A., Cambridge University Press: 2015; 180–196.

[21] Drexler W. and Fujimoto J.G. State-of-the-art retinal optical coherence tomography', Progress in retinal and eye research, 2008; 27 (1) pp.45–88. doi: 10.1016/j.preteyeres.2007.07.005 18036865 10.1016/j.preteyeres.2007.07.005

[22] Winn B., Whitaker D., Elliott D.B. and Phillips N.J. Factors affecting light-adapted pupil size in normal human subjects', Investigative ophthalmology & visual science, 1994; 35 (3), pp.1132–1137. 8125724

[23] Verriest G. Further studies on acquired deficiency of color discrimination. Journal of the Optical Society of America, 1963; 53 pp.185–195. 13996879 10.1364/josa.53.000185

[24] Chylack L.T. Jr., Wolfe J.K., Singer D.M., Leske M.C., Bullimore M.A., Bailey I.L., et al The Lens Opacities Classification System III. The Longitudinal Study of Cataract Study Group. Archives of ophthalmology (Chicago, Ill.: 1960), 1993; 111 (6), pp.831–836. 10.1001/archopht.1993.01090060119035 8512486

[25] Olafsdottir E., Andersson D.K. and Stefansson E. The prevalence of cataract in a population with and without type 2 diabetes mellitus. Acta Ophthalmologica, 2012; 90 (4), pp.334–340. doi: 10.1111/j.1755-3768.2011.02326.x 22176834 10.1111/j.1755-3768.2011.02326.x

[26] Roy M. S., McCulloch C., Hanna A. K. and Mortimer C. Color vision in longstanding diabetes mellitus. Br. J. Ophthalmol., 1984; 68, 215–217. 6696875 10.1136/bjo.68.3.215 PMC1040290

[27] Ismail G.M. & Whitaker D. Early detection of changes in visual function in diabetes mellitus. Ophthalmic and Physiological Optics, 1998; 18, 3–12. 9666905

[28] Barton F.B., Fong D.S., Knatterud G.L. & ETDRS Research Group. Classification of Farnsworth-Munsell 100-Hue Test Results in the Early Treatment Diabetic Retinopathy Study. American Journal of Ophthalmology, 2004; 138, 119–124. doi: 10.1016/j.ajo.2004.02.009 15234290 10.1016/j.ajo.2004.02.009

[29] O’Neill-Biba M., Sivaprasad M., Rodriguez-Carmona S., Wolf M., J. E. and Barbur J. L. Loss of chromatic sensitivity in AMD and diabetes: a comparative study. Ophthal. Physiol. Opt., 2010; 30: 705–716. 10.1111/j.1475-1313.2010.00775.x 20883358

[30] Barber A.J., Lieth E., Khin S.A., Antonetti D.A., Buchanan A.G. and Gardner T.W. Neural apoptosis in the retina during experimental and human diabetes. Early onset and effect of insulin. The Journal of clinical investigation, 1998; vol. 102, no. 4, pp. 783–791. doi: 10.1172/JCI2425 9710447 10.1172/JCI2425 PMC508941

[31] Barber A.J., Gardner T.W. and Abcouwer S.F. The significance of vascular and neural apoptosis to the pathology of diabetic retinopathy. Investigative ophthalmology & visual science, 2011; vol. 52, no. 2, pp. 1156–1163. 21357409 10.1167/iovs.10-6293 PMC3053099

[32] Nishikawa T., Edelstein D., Du X.L., Yamagishi S., Matsumura T., Kaneda Y., et al Normalizing mitochondrial superoxide production blocks three pathways of hyperglycaemic damage. Nature, 2000; 404 (6779) pp.787–790. doi: 10.1038/35008121 10783895 10.1038/35008121

[33] Hammes H.P., Du X., Edelstein D., Taguchi T., Matsumura T., Ju Q., et al Benfotiamine blocks three major pathways of hyperglycemic damage and prevents experimental diabetic retinopathy. Nature medicine, 2003; 9 (3) pp.294–299. doi: 10.1038/nm834 12592403 10.1038/nm834

[34] Barber A. J. A new view of diabetic retinopathy: a neurodegenerative disease of the eye. Prog. Neuropsychopharmacol. Biol. Psychiatry, 2003; 27, 283–290. doi: 10.1016/S0278-5846(03)00023-X 12657367 10.1016/S0278-5846(03)00023-X

[35] Seigel G.M., Chiu L., Paxhia. Inhibition of neuroretinal cell death by insulin-like growth factor-1 and its analogs. Molecular vis., 2000; 6: 157–163. 10973501

[36] Barber A.J., Nakamura M., Wolpert E.B., E C, Seigel G.M., Reiter Antonetti D.A. Insulin rescues retinal neurons from apoptosis by the phosphatidylinositol 3-kinase/Akt-mediated mechanism that reduces the activation of caspase-3. J Biol Chem., 2001; 276 (35): 32814–32821. doi: 10.1074/jbc.M104738200 11443130 10.1074/jbc.M104738200

[37] Gariano R.F., Gardner T.W. Retinal angiogenesis in development and disease’, Nature, 2005; 438 (7070): 960–966. doi: 10.1038/nature04482 16355161 10.1038/nature04482

[38] Dean F.M., Arden G.B. and Dornhorst A. Partial reversal of protan and tritan colour defects with inhaled oxygen in insulin dependent diabetic subjects. The British journal of ophthalmology, 1997; 81 (1) pp.27–30. 9135404 10.1136/bjo.81.1.27 PMC1721986

[39] Connolly D.M., Barbur J.L., Hosking S.L. and Moorhead I.R. Mild hypoxia impairs chromatic sensitivity in the mesopic range. Investigative ophthalmology & visual science, 2008; 49(2): 820–827. 18235033 10.1167/iovs.07-1004

